# The General Principle of the Warburg Effect as a Possible Approach for Cancer Immunotherapy: The Regulatory Effect of Plant Extracts Could Change the Game

**DOI:** 10.3390/molecules30020393

**Published:** 2025-01-18

**Authors:** Donika Ivanova, Severina Semkova, Boncho Grigorov, Milena Tzanova, Ana Georgieva, Dancho Danchev, Biliana Nikolova, Zvezdelina Yaneva

**Affiliations:** 1 Department of Pharmacology, Animal Physiology Biochemistry and Chemistry, Faculty of Veterinary Medicine, Trakia University, 6000 Stara Zagora, Bulgaria; zvezdelina.yaneva@trakia-uni.bg; 2 Department of Chemistry and Biochemistry, Faculty of Medicine, Trakia University, 6000 Stara Zagora, Bulgaria; 3 Department of Electroinduced and Adhesive Properties, Institute of Biophysics and Biomedical Engineering, Bulgarian Academy of Sciences, 1113 Sofia, Bulgaria; biliananikolova2000@yahoo.com; 4 Department of Molecular Biology, Immunology and Medical Genetics, Faculty of Medicine, Trakia University, 6000 Stara Zagora, Bulgaria; boncho.grigorov@trakia-uni.bg; 5 Department of Biological Sciences, Faculty of Agriculture, Trakia University, 6000 Stara Zagora, Bulgaria; milena.tsanova@trakia-uni.bg; 6 Trakia University, 6000 Stara Zagora, Bulgaria

**Keywords:** cancer, Warburg effect, redox metabolism, macrophage polarization, natural plants

## Abstract

The interpretation of the biochemistry of immune metabolism could be considered an attractive scientific field of biomedicine research. In this review, the role of glycolysis in macrophage polarization is discussed together with mitochondrial metabolism in cancer cells. In the first part, the focus is on the Warburg effect and redox metabolism during macrophage polarization, cancer development, and management of the immune response by the cancer cells. The second part addresses the possibility of impacts on the Warburg effect through targeting peroxisome proliferator-activated receptors (PPARs). This could be an activator of native immune responses. Because of the reported serious adverse effects of using synthetic ligands for PPARs in combination with chemotherapeutics, searches for less toxic and more active PPAR inhibitors, as well as blocking undesirable cellular PPAR-dependent processes, are in progress. On the other hand, recent research in modern immunotherapy has focused on the search for gentle immune-modulating natural compounds with harmless synergistic chemotherapeutic efficacy that can be used as an adjuvant. It is a well-known fact that the plant kingdom is a source of important therapeutic agents with multifaceted effectiveness. One of these is the known association with PPAR activities. In this regard, the secondary metabolites extracted from plants could change the game.

## 1. Introduction

Since the critical role of polarized macrophages in supporting the development of malignant diseases has recently been well described, the focus on immunometabolism is considered an attractive field of modern biomedical science. There is scientific agreement on the fact that mitochondrial metabolism plays a pivotal role in the regulation of the processes of cell survival, differentiation, and cell death. At the same time, the modulation of mitochondrial function is tightly connected to alteration in immune cell polarization. The discussion that the mitochondrial metabolism of immune cells has a fundamental role during their transformation from a state of relative metabolic quiescence to a highly active metabolic state during the activation phase of immune responses could serve as a platform for deep investigations [1,2].

## 2. The Role of Glycolysis and Oxidative Phosphorylation in the Process of the Activation Phase of Immune Responses

**Figure 1:**
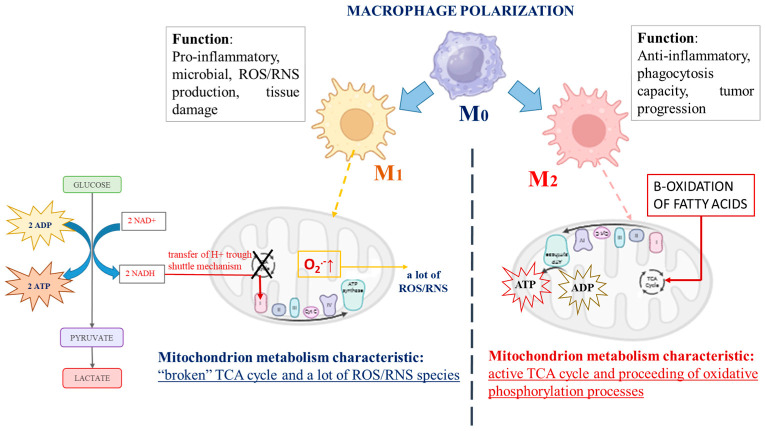
The schematic presentation of the main differences in biochemical profiles and functions of both types of immune-activated polarized macrophages.

There are a number of studies indicating the capacity of macrophages to switch their metabolic profile with remarkable plasticity, depending on the environment [3,4]. During immune stimulus, macrophages switch from a quiescent (non-polarized) state, called “M0 macrophages”, to two distinct activated states, described as “classically activated” M1 macrophages or “alternatively activated” M2 macrophages, which could progress into each other [3,5,6]. An interesting fact is that cancer cell mitochondrial metabolism is similar to that described for M1 polarized macrophages, although, in strongly hypoxic regions of advanced cancers, plenty of M2 polarized macrophages have been observed. M1 and M2 macrophages possess totally different biochemical metabolic profiles depending on their polarization [7], as schematically represented in Figure 1. The role of M1 macrophages is to provoke the production of high levels of pro-inflammatory cytokines, provoke large amounts of reactive oxygen and/or nitrogen species (ROS/RNS), and display efficient microbial properties. By contrast, M2 macrophages exhibit anti-inflammatory functions and are involved in tissue remodeling, helminth infections, and the promotion of tumor growth [8]. Biochemically, the distinction between M1 and M2 macrophages is focused on mitochondrial energy production and the expression of their glycolytic profile [9]. The main factor impacting the energy supply mode of M1 macrophages is glycolysis, the intracellular energy production method, in an oxygen-independent manner. In contrast, M2 macrophages are characterized by the proceeding of oxidative phosphorylation processes and fatty acid oxidation, which are their typical mechanisms for energy provision [8,10,11,12,13,14,15]. As proof, Jha et al. reported systemic changes during murine macrophage M1 and M2 polarization and observed “broken” cycles of tricarboxylic acids (TCA cycle) in M1 macrophages. According to the authors, the fragmentation of the TCA cycle could be provoked by reduced activity of the isocitrate dehydrogenase enzyme (an enzyme that converts isocitrate to alpha-ketoglutarate in the Krebs cycle). To compensate for the lower cellular energy levels, the alternative activation of the aspartate-argininosuccinate shunt (the linking metabolite cycle between the TCA cycle and urea cycle) could be activated. On the other hand, aspartate depletion leads to the inhibition of the enzyme activity of aspartate aminotransferase, blocking of the urea cycle, and encouragement of mitochondrial respiration processes [16]. Mills et al. discussed the central role of mitochondria during the macrophage’s differentiation processes. The authors reported a changed mitochondrial function and shift from the oxidative phosphorylation of ATP production to glycolysis when macrophages were stimulated by lipopolysaccharide. Their explanation is associated with the passing of electrons through the succinate dehydrogenase complex (the second protein in the mitochondrial respiratory chain) instead of the NADPH/ubiquinone oxidoreductase (first mitochondrial respiratory chain complex) after stimulation of macrophages, which is associated with the generation of a large amount of ROS in these cells and the initiation of inflammation [17]. Vats et al. also published similar results. According to them, the activated M2 macrophages displayed elevation in the levels of ATP production as a result of β-oxidation of fatty acids and “broken” mitochondrial metabolism [12]. Almost identical biochemical metabolic changes to other immune cells have also been reported [18,19]. The effector T-cell subsets, which promote inflammation, also display a predominantly glycolytic phenotype, in contrast to the anti-inflammatory regulatory T-cell (Treg) subgroup, where mitochondrial oxidative phosphorylation has the main function in ATP production [20,21,22]. The Toll-like receptors stimulating dendritic cells have also shown encouragement of glycolysis metabolism, with the aim of supporting the production and secretion of immune regulatory cytokines, including IL-12, which promote IFN-γ production and the polarization of activated CD4 T cells [18,23].

It seems that an important role in the activation (polarization) of the immune cells is the metabolite pathway interruption between glycolysis processes in the cytosol and oxidative phosphorylation in the mitochondria. The separation between the essential metabolite pathways could be associated with the activation/inhibition of regulatory enzymes and with the changed intracellular redox homeostasis and generation of different types of ROS. Considering that glucose metabolism has a crucial role in basic cellular energy-related processes, the intercalation of alternative metabolite pathways, depending on the environmental conditions and the cellular energy-consuming needs, is highly obligatory.

## 3. The “Broken” Mitochondrial Metabolism Is an Essential Mark, Determining Similarity Between Cancer and Immune Cells

One of the general focuses of cancer cells is the excessive consumption of glucose during hypoxia, which is the familiar Warburg effect [24,25,26,27,28,29]. Due to induced genetic alterations as a result of transformed environmental conditions, different signal transduction pathways for the synthesis of molecules used for cancer growth encouragement and survival are activated in the developing cancer cells. On the other hand, accelerated proliferation, which is a characteristic mark of cancer cells, additionally stimulates hypoxic conditions [30,31,32]. Evidence has shown that malfunctioning mitochondria in cancer cells and the disrupted intracellular redox homeostasis could be a reason for cancer development, but the exact biochemical mechanism still needs to be elucidated. In this regard, data have been reported that succinate accumulation induces hypoxia [33]. Succinate dehydrogenase is an enzyme that catalyzes the oxidation reaction of succinate to fumarate in the citric acid cycle. Its activity is strongly dependent on the ratio of its cofactor FAD/FADH_2_ and the ratio of NAD^+^/NADH. If the cofactors are present predominantly in their reduced forms, the function of succinate dehydrogenase is impaired, and succinate accumulation is observed [33,34]. Tseng et al. reported on the escalation of tumor malignancy by repressing succinate dehydrogenase activity in hepatocellular carcinoma (HCC) cell lines. It provoked alteration in energy metabolism, injury of the mitochondrial membrane potential, decreased expression of complex III and IV in the electron transport chain, and the promotion of glycolysis as well as acidic status in the studied cells. On the contrary, overexpression of succinate dehydrogenase reduces glucose metabolism and lactate dehydrogenase activity [35]. Another observation has indicated that a loss of succinate dehydrogenase activity is connected with succinate accumulation and impaired mitochondrial function via induction of a pseudo-hypoxic phenotype [36]. During the investigation of biochemical kinetics reactions of succinate oxidation to fumarate, it was elucidated that the step could be evaluated as a rate-limited reaction step because it is the slowest step in the TCA cycle [37,38,39,40]. The oxidation reaction of succinate to fumarate is given below:

succinate + FAD ↔ fumarate + FADH_2_ (1)

succinate → fumarate + 2H^+^ + 2 e^−^ reducing agent (2)

(Another reducing agent in this reaction is FADH_2_ if the reaction occurs in the reverse direction.)

FAD + 2H^+^ + 2 e^−^→ FADH_2_ oxidized forms (3)

(Another oxidized agent in this reaction is fumarate if the reaction occurs in the reverse direction.)

Hypoxia-inducible factor 1-alpha (HIF-1α) triggered during hypoxia is also activated and acts to repress mitochondrial function. It provokes inhibition of the pyruvate dehydrogenase activity, interrupts the acetyl-CoA formation, and induces variation in the TCA cycle [41,42]. As a result, a reduction in the levels in the intracellular reducing equivalent NAD^+^/NADH and overexpression of the glycolysis metabolic pathway are detected.

NAD^+^/NADH is a redox couple, actively participating in glycolysis and mitochondrial oxidative phosphorylation (as a donor of electrons to complex I), and is known as a regulator of energy metabolism, DNA repair, gene expression, and stress response [43,44,45]. The elevated NAD^+^ levels enhance glycolysis via stimulation of glyceraldehyde 3-phosphate and lactate dehydrogenase activities since their catalytic activity enzymes require NAD^+^ as a co-enzyme [44,46,47]. In this regard, da Veiga Moreira et al. compared the energy status of malignant and normal (non-cancer) cells extracted from fresh human colon tissues and established a five- to ten-fold elevation of the ratio of NAD^+^/NADH and NADP^+^/NADPH in cancer cells compared to normal ones, respectively [48]. Similarly, experimental data have explained that the extreme levels of NAD^+^ detected in various tumors (including ovarian, breast, prostate, colorectal, gastric, endometrial carcinomas, melanoma, and gliomas) lead to upregulated nicotinamide phosphoribosyl transferase activity [43,49,50,51]. Conversely, downregulated nicotinamide phosphoribosyl transferase suppresses tumor cell growth both in vitro and in vivo due to the depletion of NAD^+^, followed by a reduction in tumor growth and induction of apoptosis [43,44,52,53,54].

**Figure 2:**
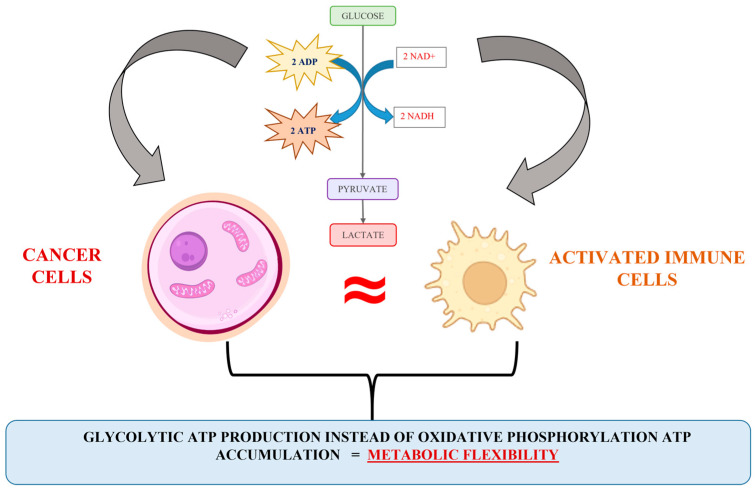
Illustration of the analogy in the cancer and activated immune cell energy metabolism.

Briefly, diverse scientific evidence has indicated that upon hypoxic stress, glycolytic ATP production provides metabolic flexibility. Meanwhile, a number of studies have indicated that glycolytic ATP production is a more accelerated process compared to oxidative phosphorylation [39,40], and a more advantageous energetic solution for proliferating cells is the synthesis of glycolytic enzymes rather than the steering of the oxidative phosphorylation machinery [35,39]. Besides, the expression of glycolytic phenotype predisposes the accumulation of precursors for synthesis processes (for example, the activation of a pentose-phosphate metabolite pathway and nucleotide synthesis), used to assist the cell proliferation pathway. Obviously, the escalation of glycolysis and the repression in normal mitochondrial activity can be recognized as a typical signal for cancer cell proliferative activity upregulation. However, an interesting analogy between the energy metabolism of cancer and activated immune cells can be observed (Figure 2). The biochemical similarity includes all Warburg effect’s points, such as hypoxia, accelerated glycolysis, lactate accumulation and established acidosis environment, elevated ROS production, changes in the availability of TCA intermediates (succinate, fumarate, and α-ketoglutarate), variation in intracellular redox homeostasis (NAD^+^/NADH; NADP^+^/NADPH), and inhibition of key enzyme complexes (pyruvate dehydrogenase, succinate dehydrogenase, etc.). It seems that the mechanism connected to the cellular energy production in cancer and activated immune cells is similar and could be discussed as a good reason for the impossibility of identifying cancer cells and escaping the native immune response.

## 4. Cancer Cells Successfully Manage Native Immune Response Through Intracellular Signals Induced by the Warburg Effect

Various studies have reported that the proliferative activity frequency of cancer cells and metastasis formation are significantly affected by the metabolic tumor microenvironment [55,56]. The macrophages, effector T cells, natural killer cells, and dendritic cells form the tumor microenvironment. They have an impact on cancer development via the secretion of different inflammatory cytokines [57,58]. For example, although macrophages derived from healthy or inflamed tissues could assist in the lysing of tumor cells through expressing immunostimulatory cytokines and tumor-associated antigens, which stimulates the proliferation and antitumor function of T-cells and natural killer (NK) cells in vitro, tumor-associated macrophages (TAMs) do not show such activities [59,60]. It seems that TAMs are impacted by some metabolites that are common for cancer and immune cells, such as the accumulation of lactate, intracellular acidosis, and induction of hypoxia [61,62]. The changed intracellular metabolism and the efflux of lactate to the extracellular space through the lactate shuttle, driven by a concentration/pH gradient or by the cellular redox state, are the possible reasons for the sustaining of continuously high rates of glycolysis [63]. Consequently, the latter leads to enhanced hypoxia, promotes acidosis in the tumor microenvironment, encourages angiogenesis, stimulates metastasis, and causes immunosuppression [64]. The role of immune system dysfunction in cancer is currently well recognized [65,66,67,68]. Altered macrophage plasticity and polarization, normally communicating with the activation of the innate immune response, can contribute both to malignancy development and to tumor vascularization [64,69,70]. In the early stage of tumor development, M1 macrophages may infiltrate, activated in response to inflammatory mediators, and release pro-inflammatory cytokines and chemokines in order to attract and encourage the development and differentiation of T-helper cells (Th1 and Th17) and NK cells [71]. In contrast, in more advanced tumors or in hypoxic regions of the tumor microenvironment, a progression of M1 to M2 macrophages has been shown [71,72,73]. As confirmation, Lin et al. demonstrated that because of lactate accumulation, activation of human macrophages to an M2 phenotype was observed. The discussed mechanism is focused on the stimulation of the Notch signaling cascade, leading to the secretion of the Chemokine (C-C motif) ligand 5 (CCL5), which has been associated with an increase in cell migration and the induction of cancer cell epithelial to mesenchymal transition in a breast cancer cell model [74]. The triggering of the ERK/STAT3 signaling pathway by a high lactate concentration, which induces M2 macrophage polarization in a breast cancer model, has also been reported [75]. Certainly, the progression of M1 to M2 macrophages affects the function of other immune cells and the tumor microenvironment. Depending on the polarization of macrophages, the plastic population of T-helper cells also exhibits a critical function in tumor immunological responses and is associated with increasing tumor growth or induced tumor suppression [76,77]. Th1 cells produce interferon gamma (IFN-γ), interleukin (IL)-2, IL-12, and tumor necrosis factor α (TNFα) cytokines, which are involved in cell-mediated pro-inflammatory responses. Th1-associated cytokines exhibit potent antitumor effects via the activation of CD8+ cytotoxic T lymphocytes and NK-mediated cytotoxicity, as well as by the upregulation of major histocompatibility complex (MHC) expression on antigen-presenting cells. Conversely, Th2 cells secret IL-4, IL-5, Il-6, IL-10, and IL-13 cytokines, which mediate the anti-inflammatory humoral response and induce immune suppression via the inhibition of Th1 cytokine production [76,78].

Due to the switched glucose metabolism, which is typical of cancer cells, the latter successfully create a mechanism by which they activate, modify, and “instruct” the immune cells in the tumor microenvironment to encourage malignancy development [63]. However, the proceeding of a glycolysis metabolite pathway in immune cells is also a classical approach used for the activation of the native immune response [79,80]. Apparently, the common intracellular metabolite characteristics of activated immune cells and cancer cells are strongly implicated in the arrest of the antitumor immune response. Thus, it could be concluded that in the “war” between the native immune response and cancer development, the forces are balanced. Additionally, the avoidance of native immune response and/or induction of apoptosis signals, specific for cancer cells, are also provided.

In the elaboration of a new approach to cancer treatment, the central role needs to be given to the immune system, since it has the great potential to destroy cancer cells without being toxic to healthy tissue and organs [81,82,83]. At the same time, one of the essential questions is how to modulate the immune system without causing potential adverse effects associated with an overreactive immune system. It has been reported that serious autoimmune disorders develop in some patients with cancer who have been tested with immune checkpoint inhibitors of PD-1 and the cytotoxic T-lymphocyte antigen 4 (CTLA4) [84]. In this regard, the targeting of specific metabolic changes, which seems to be similar for cancer and TAMs, could be a possible way to achieve cancer recognition and “unlocking” of the native immune response. However, clinical applications and safety profiles should always be considered. For example, emerging issues such as immune-related liver adverse events and drug-induced autoimmune hepatitis, as well as the role of toxicants and drugs in the development of autoimmune hepatitis and disease relapse, should be discussed [85,86].

## 5. The Impact of the Warburg Effect Could Be a Possible Approach to Development of Cancer Immunotherapy

**Figure 3:**
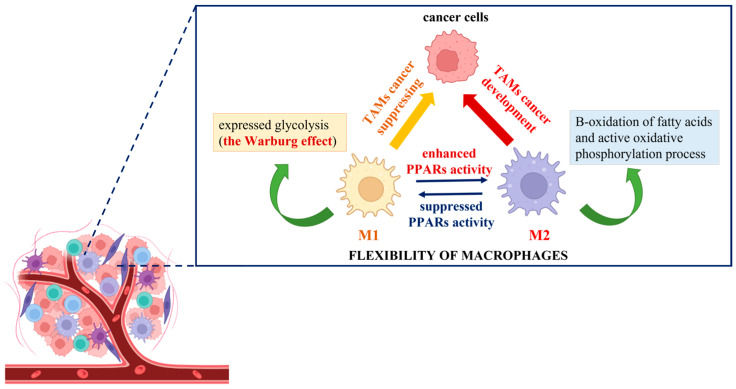
Schematic presentation of the cancer immunotherapy switch hypothesis based on the “Warburg effect” shift.

The elaboration of new anticancer approaches using the Warburg effect principle could provide an adequate way to impact tumor-associated macrophages (TAMs) and affect malignancy development. To demonstrate the different responses of cancer cells co-cultured with polarized M1 and M2 macrophages and consequently treated with etoposide, Genin et al. designed a convenient model for human macrophage polarization using the human monocytic cell line THP-1. The researchers established an increase in apoptosis signals in cancer cells in the presence of THP-1 M1-polarized macrophages, in contrast to M2 THP-1 macrophages, where a protective effect was observed [87]. This is the first experimental demonstration that THP-1 polarized macrophages display functions similar to the ones described for polarized TAMs, and it could be used as proof of their flexibility and adaptability to extrinsic stimuli. In this regard, the novel anticancer strategies could be associated with targeting TAMs and inhibition of the M2 phenotype or induction of M2-to-M1 reprogramming together with the initiation of cancer cell death [88]. The utilization of knowledge about the Warburg effect is at the center of the conception of complex cancer immunotherapy. In the search for identical mechanism(s) by which modulation of TAMs, regulation of the innate immune system function, and induction of cancer cell death could be achieved, spotlights have been focused on peroxisome proliferator-activated receptors (PPARs). Generally, they are ligand-dependent transcription factors that influence several signal transduction pathways, including DNA repair, glucose metabolism, lipid metabolism, intracellular redox homeostasis, and insulin sensitization [89,90,91]. The molecular mechanism of PPAR activation includes the post-translational modification of proteins (PARylation) where the branched ADP-ribose unit(s) are synthesized, and NAD^+^ is used as donor molecules [92,93]. As of present, reports about the activation of M2 polarized macrophages, supported by enhanced PPAR activity, can be easily found in the literature. For example, in their experiments with isolated human monocytes, Bouhlel et al. noted that in the presence of an appropriate M2 stimulus, such as IL-4, macrophages displayed upregulation of PPARγ activity and thus enhanced expression of the M2 phenotype [94]. Daniel et al. also established that upon repeated stimulation with interleukin (IL)-4, bone marrow-derived macrophages increased their PPARγ protein levels in cytosol, which is connected with a powerful expression of the M2 phenotype. Moreover, during muscle regeneration in a mouse model of injury, the detection of IL-4 and PPARγ in the affected tissue was observed [95]. Other studies have indicated that IL-4/IL-13 strongly increases the production of different endogenous PPAR ligands (13-HODE, 15-HETE, and 15d-PGα) and PPAR coactivators (PGC-1), thereby stimulating PPAR transactivating activity [12,96,97]. As mentioned above, polarized M2 macrophages obtain energy from fatty acid oxidation and oxidative phosphorylation due to the intact TCA cycle, compared to M1 macrophages, where it is due to “broken” mitochondrial metabolism, glycolysis, and the pentose phosphate pathway [9]. For example, Keller et al. established that the physiological concentrations of polyunsaturated fatty acids activated PPARα and, in combination with the retinoid X receptor beta (RXR-beta), upregulated the acyl-CoA oxidase gene promoter and enhanced the peroxisomal-β-oxidation pathway by impacting a rate-limiting enzyme [98]. Additionally, other experiments have indicated that the activated PPARs modulate the gene expression of several enzymes that participate in a catabolic process of mitochondrial β-oxidation of fatty acids. For example, Barrero et al. showed that the human carnitine palmitoyltransferase gene, which controls the expression of the enzyme catalyzing the primary rate-controlling step in fatty acid oxidation, is a target of PPARα [99]. During an in vivo experiment, Odegaard et al. made the conclusion that the monounsaturated fatty acids activate PPARδ and enhance the maturation of alternatively activated macrophages in tissues. This suggests that the increasing concentration of cytosolic free fatty acids could promote the polarization of alternatively activated M2 macrophages through enhanced PPAR activation [100]. As a possible approach for the reversible programming of TAMs, the switch of the cellular metabolite from M2 to M1 phenotype can be considered. This process represents a metabolic change from increased oxidation of fatty acids to highly pronounced glycolysis (Warburg effect). It is initiated by suppressing the activity of PPARs and may act to support a reduction in cancer cell growth and development (Figure 3).

Moreover, the expression of PPARs in various types of cancer cells has also been reported [101]. It is not a coincidence that different clinical and preclinical data have described therapies for solid tumors with synthetic ligands for PPARs, such as thiazolidinediones, applied individually or in combination with chemotherapeutic agents [102,103,104,105]. Thiazolidinediones, also called “glitazones”, are medicaments used to improve insulin-dependent glucose uptake during the treatment of type 2 diabetes. They affect the intracellular metabolic pathway to increase insulin receptor sensitivity and decrease hepatic gluconeogenesis [106]. It seems that thiazolidinediones are appropriate adjuvants in cancer therapy, but if they are administered in the long term, concerning side effects, such as body weight gain, anemia, osteoporosis, fluid retention, developing congestive heart failure, increased risk of myocardial infarction, and consequently, cardiovascular death, can be observed [107,108,109]. Searches for less toxic and more active PPAR inhibitors, as well as for blocking undesirable cellular PPAR-dependent processes, are still in progress. In the past decade, the focus has switched from synthetic to natural compounds due to their higher bioavailability, biocompatibility, and biodegradability [110]. It is a well-known fact that the antiviral, antibacterial, and antitumor activity of various natural plant metabolites adds to their high antioxidant potential. Moreover, there is evidence that some natural compounds affect PPAR-mediated signaling pathways [111,112]. Since ancient times, the immunomodulating capacity of numerous medical plants has been known and researched. However, their potential to influence inflammatory processes due to the modulation of both innate and adaptive immune responses is still being investigated [113,114].

## 6. The Regulatory Effect of the Secondary Metabolites of Plant Extracts Could Change the Game

**Figure 4:**
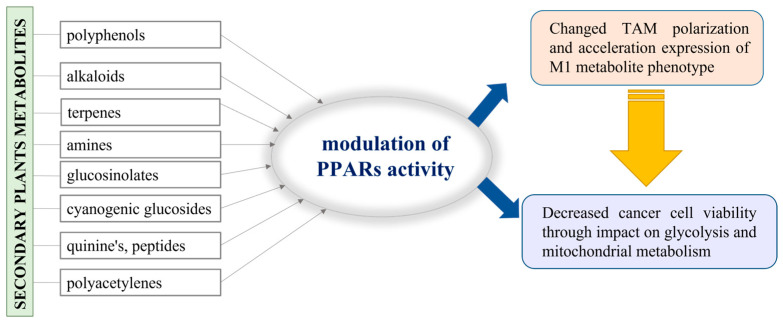
Schematic presentation of the possible application of the plant metabolites in PPAR activity modulation.

As typical sessile organisms, plants are provoked to elaborate a mechanism by which to protect themselves from various environmental challenges and to resist evolutionary changes, generally called “the natural selection”. The secondary metabolites produced by plants do not have a role in their basic life processes, but they ensure the competitiveness and survival of plants under stress conditions and play a vital role in adaptation and defense against pests [115]. For example, the variety of polyphenols identified in plants as secondary metabolites are generally used for self-protection against UV radiation and plant pathogen invasion [116]. In addition to polyphenols, numerous bioactive compounds, such as alkaloids, terpenes, amines, glucosinolates, cyanogenic glucosides, quinones, peptides, and polyacetylenes, are also recognized as secondary plant metabolites [117]. In fact, plants provide this important resource of substances with proven beneficial therapeutic effects, so it is not surprising that they have been extensively studied in both scientific and industrial fields. Additionally, there is information that some of them have epigenetic activity and could be involved in the regulation of gene expression, including PPAR-dependent genes [118]. Hence, we may consider a possible approach where the secondary metabolites in plants are involved in the alteration of TAM polarization and impact cancer cell viability (Figure 4).

Data have indicated that honokiol, a natural biphenolic compound, derived from the stem and bark of the traditional Chinese herbal drug *Magnolia officinalis*, can bind to a PPARγ ligand-binding domain, stimulate glucose uptake in 3T3-L1 adipocytes, and prevent hyperglycemia in diabetic KKAy mice [119]. The anti-tumor activity of honokiol has been reported in different types of cancers, such as colorectal, breast, gastric, and hepatocellular carcinoma [120,121,122]. For example, it has been described that honokiol induced apoptosis and markedly downregulated the expression of peroxisome proliferator-activated receptor-gamma (PPARγ) and COX-2 in different human gastric cancer cells (AGS, MKN45, N87, and SCM-1) and tumors of xenograft mice [121]. Recently, an in vitro and in vivo study reported that honokiol, packed in liposome nano-carriers, affected macrophage polarization and impacted the progression of glioblastoma. The data from this study showed the suppression of cancer growth through an impact on TAMs, inhibition of M2, and promotion of M1 polarization of macrophages [123]. This presents strong evidence regarding the influence of plant secondary metabolites on TAM polarization through the modulation of PPARs and repression of malignancy development.

Recently, Rutkowska et al. published a comprehensive review of molecular mechanisms, pharmacokinetics, toxicology, and plant sources of Juglanin—an extract from a widespread plant species from divergent botanical families [124]. In the paper, 47 articles are cited, discussed, and listed as examples of studying the multifaceted biological activity of juglanin in various diseases and pathologies (from fibrosis to cancer). Moreover, juglanin, a flavonol extracted by the widespread plant *Polygonum aviculare*, has been applied on UVB-stimulated B16F10 melanoma cells and demonstrated a dose-dependent decrease in cell viability after 24 h incubation, as well as the induction of apoptosis via promotion of poly (ADP-ribose) polymerase (PARP) cleavage [125]. The anti-tumor effect of juglanin has been demonstrated on human leukemia HL-60 cells, where, along with the initiation of mitochondrial-dependent apoptosis pathways of cell death, fragmentation of PARP was also registered [126].

As described above, activated PPARs are connected with the promotion of fatty acid oxidation in cells, but the treatment of palmitate-stimulated HK2 cells with juglanin has shown the protective effect of the studied flavanol through lipid accumulation reduction and repressed inflammation in those cells [127]. Other research studies have also described the anti-inflammatory role of juglanin [128,129].

Rosemary (*Rosmarinus officinalis* L.), an evergreen shrub that grows along the Mediterranean Sea, has displayed significant antimicrobial, anti-inflammatory, anti-oxidant, anti-apoptotic, anti-tumorigenic, antinociceptive, and neuroprotective properties [130]. The secondary metabolites established in a volatile fraction of the rosemary extract include different types of flavonoids, phenolic acids, diterpenoids, triterpenoids, and lignans, but the phenolic compound carnosic acid is detected in a predominant concentration [131]. It has been observed that carnosic acid and gallic acid, another phenolic acid detected in a lower concentration in the rosemary extract, displayed selective cytotoxic activity along with inhibitory effects on poly (ADP-ribose) polymerase against BRCA2-deficient Chinese hamster lung V-C8 cells [132]. There is information that a deficiency of the human tumor suppressor gene BRCA2 is a genetic mutation frequently connected with an increased risk of hereditary breast cancer development [133]. A number of studies have reported on the effectiveness of rosemary extracts on PPAR. For example, Rau et al. proposed a specific model of action for carnosol and carnosic acids (components of rosemary extracts) based on PPARγ’s agonistic activity [134]. The authors also explained the anti-inflammatory and antiproliferative effects of the extract, with the documented glucose-lowering potential attributed to PPARγ activation. Tu et al. demonstrated increased glycolysis and fatty acid oxidation via activation of PPARγ pathways and AMP-activated protein kinase (AMPK) in HepG2 cells after treatment with rosemary extract [135]. The anti-inflammatory potential of the phenolic compounds contained in *Rosmarinus officinalis* L. was also demonstrated in a study by Schwager et al., where after treatment with natural compounds, the alteration of the activity of genes appeared in a pattern of acute and chronic inflammatory processes [136].

The second metabolites contained in the medical plant *Cannabis sativa* L. represent a unique class of terpenophenolic compounds called cannabinoids, which have been extensively explored for the treatment of various diseases [137]. It is a well-described fact that CBD (one of the main phytocannabinoids) is a PPARγ agonist and changes its expression. Many biological effects of CBD (on the immune system, cancer, vasculature, adipose tissue, etc.) are mediated at least partially via PPARγ activation, and they are found to be time- and tissue-dependent [138]. Moreover, the in vitro activation of the cannabinoid receptor 1 (CB1) on macrophages in colorectal cancer indicated the suppression of M2 macrophage polarization and decreased the proliferation, migration, and invasion of the tested human colorectal cancer cell lines, while silencing of CB1 promoted M2 polarization and cancer cell proliferation [139].

A number of popular secondary plant metabolites have also been investigated as natural PPAR agonists in the treatment of hepatocellular carcinoma. There is information that some of them could be applied in a targeted therapy [140,141]. The anticancer activity and the protective effect of curcumin [142,143], resveratrol [144,145,146], and epigallocatechin gallate [147] in cancers could be associated with the targeting of PPARs by which the compounds regulate glucose and lipid metabolism.

## 7. Conclusions

Targeting the unique biochemical immune metabolism appears to be a promising therapeutic strategy for various inflammatory diseases, including cancer.

The ability of immune cells to demonstrate flexibility depending on the need for pro-inflammatory or anti-inflammatory activity has long been a subject of study and is still under investigation. Since most synthetic chemotherapeutic drugs are immunosuppressant, the elaboration of a new approach, based on the improvement of the modulatory function of the immune system, is still widely discussed. However, the risk of serious autoimmune disorders associated with an overreactive immune system has been reported [84]. In this regard, the focus of modern cancer immunotherapy is to search for a synergistic effect between less toxic chemotherapeutic drugs and compounds that gently modulate the immune system. Plant-based secondary metabolites appear to be suitable candidates. Their immunomodulatory activity has been studied since ancient times; however, modern medicine also demonstrates their ability to alter gene expression, including PPAR-mediated signal transduction pathways. Some of the above-described events due to the PARylation have an impact on intracellular redox-equivalents (NAD^+^/NADH), lipid accumulation, glucose, and mitochondrial metabolism. All of them are key aspects associated with the impact of the Warburg effect in cancer cells and with the alteration in TAM polarization. Thus, it could be suggested that the promotion of the Warburg effect may be considered a possible approach to cancer immunotherapy and a novel perspective for personalized medicine.

## Data Availability

Not applicable.
